# Testing the Utility of a Data-Driven Approach for Assessing BMI from Face Images

**DOI:** 10.1371/journal.pone.0140347

**Published:** 2015-10-13

**Authors:** Karin Wolffhechel, Amanda C. Hahn, Hanne Jarmer, Claire I. Fisher, Benedict C. Jones, Lisa M. DeBruine

**Affiliations:** 1 Center for Biological Sequence Analysis, DTU Systems Biology, Technical University of Denmark, Kongens Lyngby, Denmark; 2 Institute of Neuroscience & Psychology, University of Glasgow, Glasgow, Scotland, United Kingdom; University of Goettingen, GERMANY

## Abstract

Several lines of evidence suggest that facial cues of adiposity may be important for human social interaction. However, tests for quantifiable cues of body mass index (BMI) in the face have examined only a small number of facial proportions and these proportions were found to have relatively low predictive power. Here we employed a data-driven approach in which statistical models were built using principal components (PCs) derived from objectively defined shape and color characteristics in face images. The predictive power of these models was then compared with models based on previously studied facial proportions (perimeter-to-area ratio, width-to-height ratio, and cheek-to-jaw width). Models based on 2D shape-only PCs, color-only PCs, and 2D shape and color PCs combined each performed significantly and substantially better than models based on one or more of the previously studied facial proportions. A non-linear PC model considering both 2D shape and color PCs was the best predictor of BMI. These results highlight the utility of a “bottom-up”, data-driven approach for assessing BMI from face images.

## Introduction

Perceptions of people’s weight based on photographs of their faces are positively correlated with their body mass index (BMI, i.e., weight scaled for height), demonstrating the existence of cues of adiposity in human faces [1–3]. Moreover, people tend to judge the faces of individuals with healthy levels of adiposity (e.g., BMIs within the range the World Health Organization describes as normal, 18.5–24.9 kg/m^2^) to be more attractive and look healthier than those of individuals who are, for example, overweight [1,4]. These results suggest that facial cues of adiposity may be important for human social interaction. Consistent with this proposal, people with particularly strong aversions to cues of high levels of adiposity in opposite-sex faces have romantic partners with lower BMIs, suggesting that perceptions of facial cues of adiposity may contribute to partner choice [2].

While the studies described above investigated how facial cues of adiposity inform social judgments and partner choice, other work has investigated the information that might be conveyed by facial adiposity. For example, people rated as having higher levels of facial adiposity report more frequent illnesses [1,3], live shorter lives [5], are more likely to have hormonal profiles associated with poor reproductive health [3], and score lower on measures of both cardiovascular health [1] and psychological wellbeing [3]. Men, but not women, rated as having higher levels of facial adiposity also show weaker immune responses to a vaccine [6,7]. Importantly, at least some of these links between perceived facial adiposity and measures of actual health remain significant when controlling for BMI, suggesting that facial cues of adiposity may contain health information over and above the information that is captured by BMI alone [3].

Given the importance of facial adiposity for both social perception and as a channel through which health information is conveyed, recent work has sought to establish the extent to which facial cues of adiposity can be objectively measured. Coetzee *et al*. [8] investigated this issue in four samples of women and four samples of men (41 to 52 individuals per sample; 381 individuals in total) by examining three facial proportions (perimeter-to-area ratio, width-to-height ratio, and cheek-to-jaw-width ratio) that pilot studies suggested might covary with BMI or that were conceptually similar to those that covaried with BMI in studies of human bodies (e.g., [9]). Although some of these facial proportions were correlated with BMI in multiple samples, the strength of the relationships showed substantial variation across samples. For example, although Coetzee *et al’s* [8] results were most consistent for width-to-height ratio, r^2^ values for these correlations varied from .01 to .23, and were significant in only four of the eight samples. Other studies [10–12] investigating the relationship between BMI and width-to-height ratio have also observed considerable variation in the strength of this relationship (r^2^ values varying from .05 to .27). Moreover, a recent meta-analysis of the relationships between facial width-to-height ratio and BMI in 22 samples estimated the mean r^2^ to be .10 [13]. Together, these results suggest that facial width-to-height explains only a small amount of the variance in BMI.

Coetzee *et al*. [8] adopted a “top-down” approach to identify quantifiable cues of BMI in the face in which the existing literature was used to make *a priori* predictions about the facial parameters that might covary with BMI. An alternative approach is to use a "bottom-up”, data-driven approach in which models are built using the Principal Components (PCs) derived from objectively defined shape and color characteristics of the face images to find the model that most reliably explains the most variance in BMI. This type of bottom-up approach has for example been used successfully to identify facial characteristics associated with facial expressions and social judgments of faces [14–16]. A key strength of this bottom-up approach is that its success is not constrained by the specific parameters selected for the study.

The main aim of the current study was to investigate whether models constructed with this type of Principal Component Analysis (PCA) approach can reliably explain more of the variance in women’s BMI than the facial proportions identified by Coetzee *et al*. [1].

Previous work suggests that both 2D shape and color information in faces contain health information (e.g., [1,17,18]). Consequently, we investigated whether applying the PCA approach to facial shape and color information individually reliably predicts women’s BMI better than Coetzee *et al’s* [8] facial proportions can. We also investigated whether a PCA approach that considers information from both facial shape and color information simultaneously is better than an approach considering only facial shape or only color information. By contrast with Coetzee *et al’s* [8] study, which focused exclusively on linear relationships between facial proportions and BMI, we also considered whether including possible nonlinear relationships between facial cues and BMI increases the amount of variance in BMI that can be reliably explained by facial characteristics.

### Methods

This research was approved by University of Glasgow’s College of Science and Engineering Ethics Committee. Each participant provided written consent for their image and data to be used.

### Materials

Digital face photographs of 526 young adult white women, 524 of who reported their age (mean age = 21.3 years, SD = 2.87 years), were taken under standardized photographic conditions. All of the women were students or staff at the University of Glasgow.

Each woman first cleaned her face with hypoallergenic face wipes to remove any makeup and was photographed a minimum of 10 minutes later. Photographs were taken in a small windowless room against a constant background and under standardized diffuse lighting conditions. Participants were instructed to pose with a neutral expression. Camera-to-head distance, focal distance, and other camera settings were held constant. Participants wore a white smock covering their clothing when photographed. Photographs were taken using a Nikon D300S digital camera and a GretagMacbeth 24-square ColorChecker chart was included in each image for use in color calibration. Following previous research (e.g., [19]), face images were color calibrated using a least-squares transform from an 11-expression polynomial expansion developed to standardize color information across images [20].

In addition to being photographed, each woman’s height (M = 166.02 cm, SD = 6.33 cm) and weight (M = 64.17 kg, SD = 11.40 kg) were measured. These measurements were used to calculate each woman’s BMI (M = 23.24 kg/m^2^, SD = 3.61 kg/m^2^).

**Fig 1:**
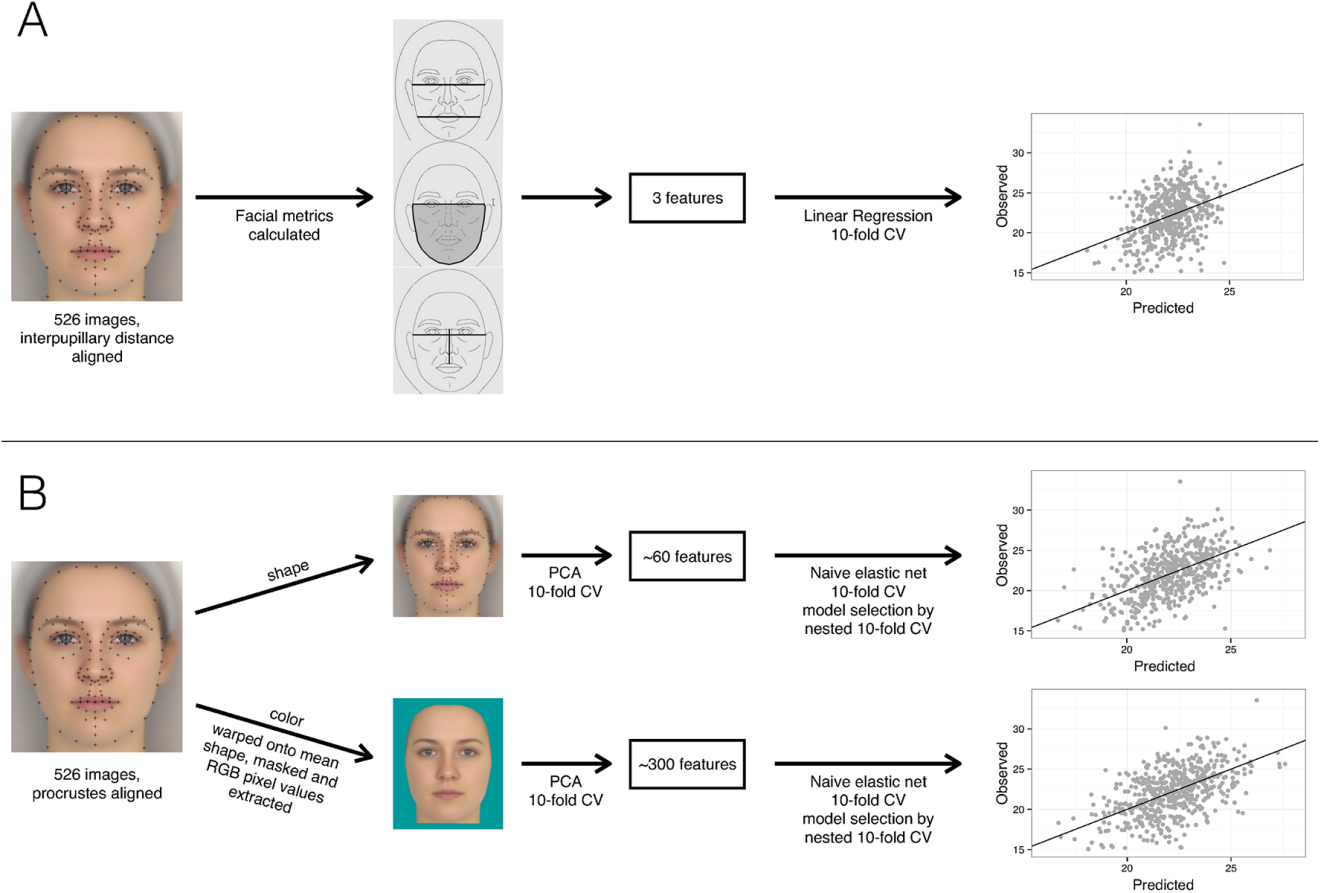
Overview of analyses. A schematic of the procedures for calculating facial metrics (A) and principal components (B) and the subsequent prediction of BMI measures.

Next, physical aspects of the face images were calculated (see Fig 1 for an overview).

### Calculating Coetzee *et al’s* [8] Facial Metrics

Following Coetzee *et al*. [8], all faces were aligned on interpupillary distance and their three facial metrics (perimeter-to-area ratio, width-to-height ratio and cheek-to-jaw width) were calculated. Following Coetzee *et al*. [8], parameter-to-area ratio was defined as the perimeter of the face (with the upper boundary defined as the line connecting the pupils) divided by the area within this perimeter. Following Coetzee *et al*. [8], width-to-height ratio was defined as the horizontal distance between the two most lateral facial points divided by the vertical distance between the most inferior point of the upper eyelid and the most superior point of the upper lip. Following Coetzee *et al*. [8], cheek-to-jaw width was defined as the horizontal distance between the two most lateral facial points divided by the horizontal distance between the lateral points of the face along the midline of the lips.

### Extracting Principal Components

**Fig 2:**
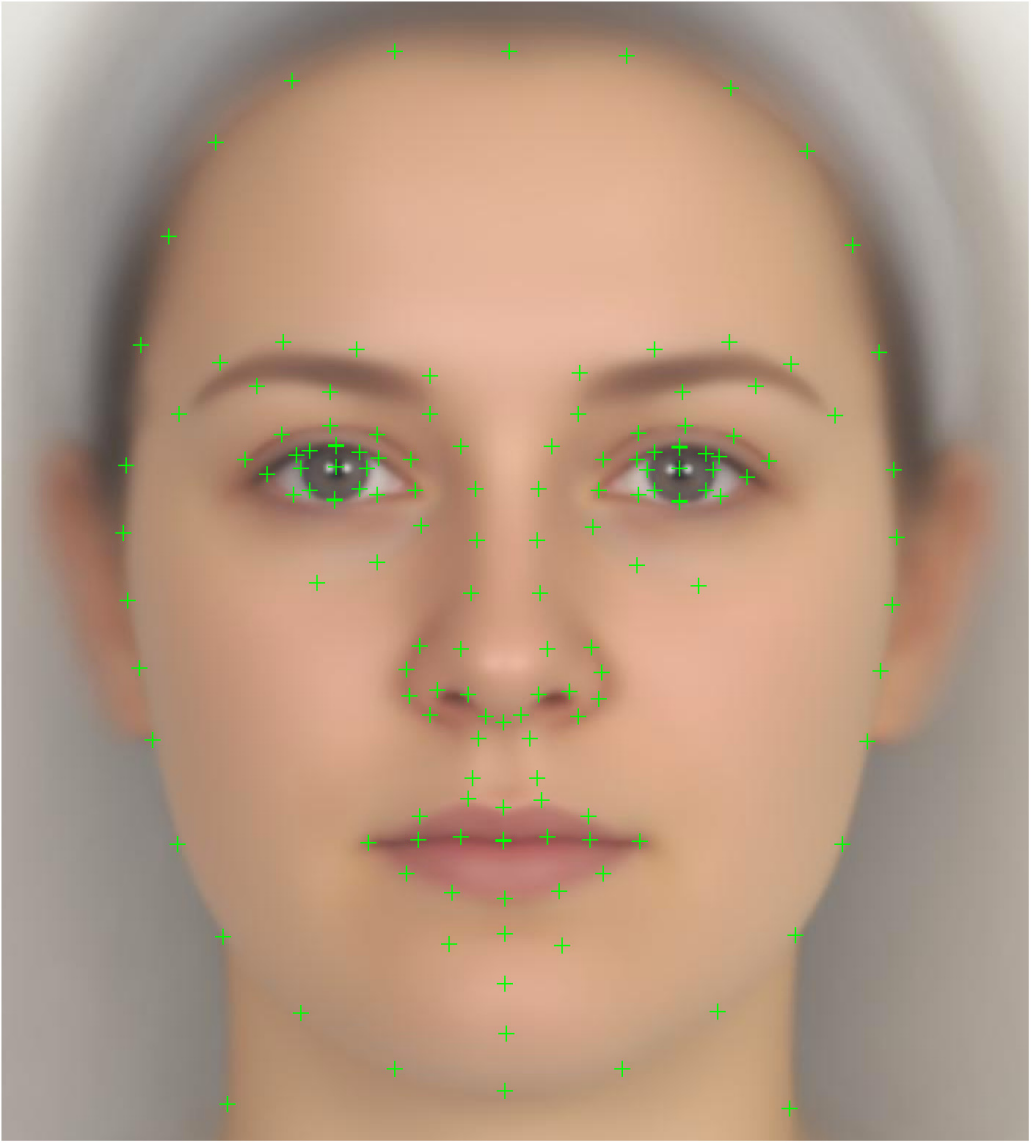
Landmark points. The 154 landmark points on the average of all 526 faces.

First, 2D face shape was defined (see Fig 2) using the same 154 landmark points commonly used to define 2D face shape and internal facial features in many previous face perception studies [21]. As is standard for this type of 2D shape analysis (e.g., [22]), faces were then procrustes aligned [23,24] to minimize the effect of translation, rotation and scaling between faces, thereby making them shape-comparable.

Next, inside a 10-fold cross-validation 2D shape PCs were calculated by a PCA on the delineation points of the faces with the number of Principal Components (PC) restricted to account for 95 percent of variance in the original data. The delineation points of the faces in the held-out fold were projected into this 2D shape PCA space. For color analyses, all faces were warped onto the mean 2D shape for the sample, using well-established methods [21]. RGB values for each pixel were extracted and inside the cross-validation a PCA was performed on these pixel values for the faces, again restricting the number of PCs to explain 95 percent of the variance. The pixel values of the faces in the held-out fold were projected into this color PCA space. Note that color PCs may capture meaningful information about 3D face shape because information about 3D face shape is represented in face images by color information.

### Statistical Analyses

All analyses were run in R v. 3.1.2 [25]. BMI measures were not normally distributed (Shapiro-Wilk normality test, p < .001), so were inverse transformed to ensure normality (Shapiro-Wilk normality test, p = .13). The set of 526 faces was divided into ten folds and models were trained by cross-validation to avoid overfitting. Inside the cross-validation, and prior to model training, BMI measures more than three standard deviations away from the mean, based on the training set, were excluded. This removed between 7 and 9 women in each fold; all excluded women were overweight with a mean BMI of 36.34 kg/m^2^ (SD = 1.98 kg/m^2^).

Cross-validation was repeated 30 times with different fold splits to ensure that results generalized across different partitioning of the data. A t-test was performed on the 30 repeats to compare model performance.

For Coetzee *et al’s* [8] facial metrics, a linear regression was used to predict BMI by repeated stratified 10-fold cross-validation. Four models with different predictors were trained: perimeter-to-area ratio only, width-to-height ratio only, cheek-to-jaw width only, and all 3 facial metrics combined. For each model, a linear regression was trained on nine folds and model performance was evaluated from the r^2^ between observed and predicted values for the held-out fold. This process was repeated until each fold had been held-out exactly once.

For the PCs, penalized regressions were used to predict BMI by repeated stratified 10-fold nested cross-validation, where the outer cross-validation estimates performance and the inner cross-validation performs model selection (described in [26]). A penalized regression uses a combination of ridge regression, which shrinks coefficients towards zero but eliminates none, and the lasso, which performs variable selection by shrinking coefficients down to zero. The amount of shrinkage is defined by the tuning parameters λ_1_ and λ_2_, where a value of zero is equal to no shrinkage. This method is called a naive elastic net and is described in greater detail in [27,28]. Inner, nested, 10-fold cross-validations were performed to find the optimal set of λ-values by doing a grid search over different combinations of λ_1_ and λ_2_-values in the range from 0 to 1000. The upper boundary of this range was found to be appropriate, since the upper limit of 1000 was never chosen as the most optimal for prediction. The inner cross-validation was repeated five times for a more robust estimation of the optimal λ-values. The chosen values were then used in the outer cross-validation and model performance was evaluated using the r^2^ between target and predicted values for the held-out fold. To explore the presence of a non-linear relationship between PCs and BMI, a support vector machine with a radial basis function kernel was also implemented [29], using the same approach and optimizing for sigma (inverse kernel width) and C (cost of constraints violation).

## Results

### Coetzee *et al’s* [8] Facial Metrics

**Fig 3:**
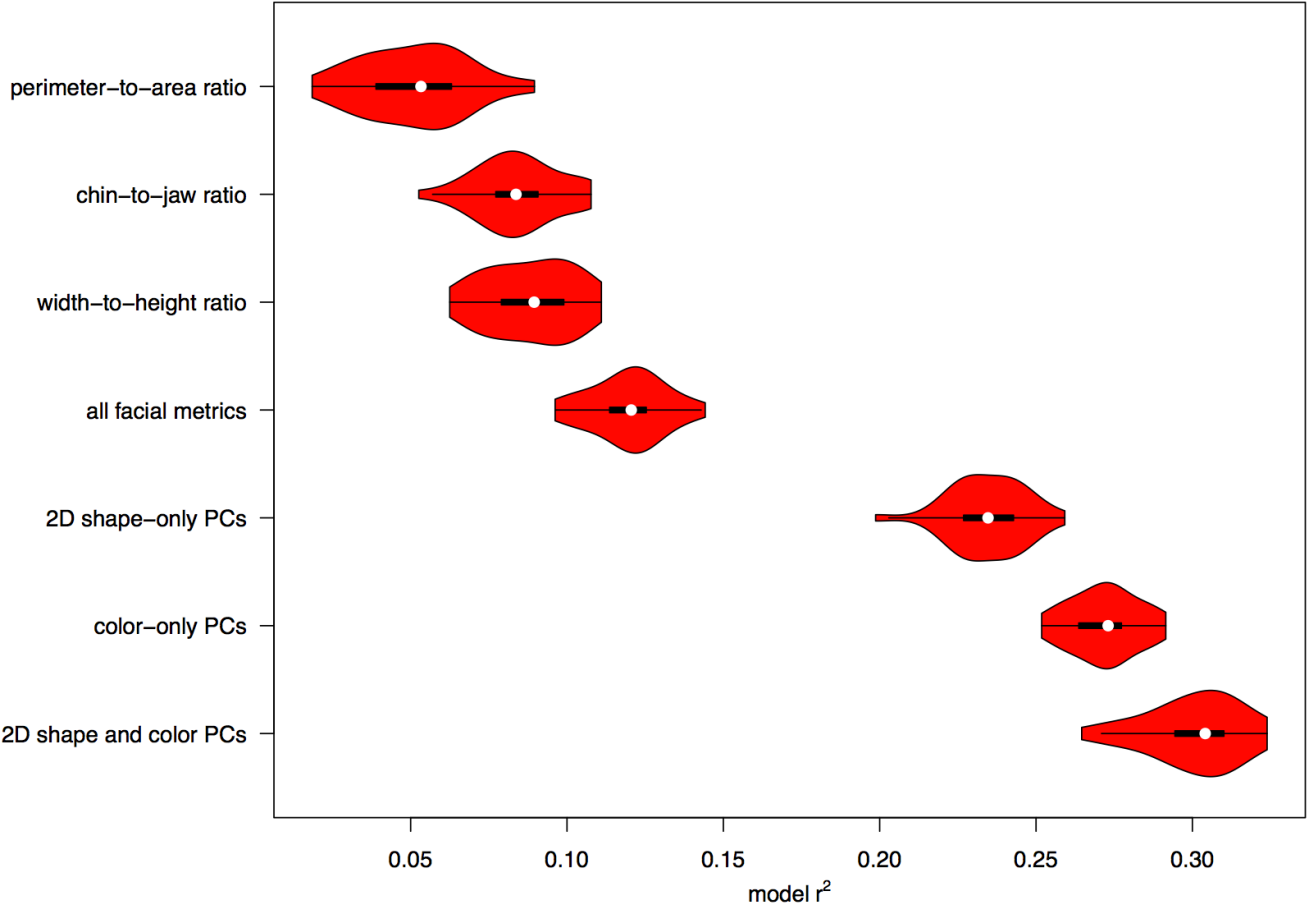
Comparison of model performance. The distribution of r^2^ values for the 30 repeats of each model. In these violin plots [30,31], the white dot shows the median value, the thick black bars span the first to the third quartiles, the whiskers span 1.5 times the interquartile range, and the red bars show the distribution of values.

The mean amount of variance in BMI (i.e., mean r^2^ across repeats) explained by each of Coetzee *et al’s* [8] facial metrics (perimeter-to-area ratio, width-to-height ratio and cheek-to-jaw width) individually ranged from 5.1% to 8.8%. The mean amount of variance in BMI explained by a regression model including all three of these metrics was 12.0%. The distribution of r^2^-values for the 30 repeats of each model are shown in Fig 3.

We compared the prediction performance of these four models (perimeter-to-area ratio only, width-to-height ratio only, cheek-to-jaw width only, and all 3 facial metrics combined) and found the combined model to be significantly better than each of the three individual models (all t(58) > 9.52, all p < .001). The model for perimeter-to-area ratio only performed significantly poorer than the other models (all t(58) < –8.55, all p < .001). The models for width-to-height ratio only and cheek-to-jaw width only performed equally well (t(58) = 1.16, p = .25).

### Principal Components

About 60 shape and 300 color PCs, each explaining diverse, holistic aspects of a female face, were extracted as described in the Methods. Due to a large number of PCs compared to the number of observations, we applied a naïve elastic net and a support vector machine with a nonlinear kernel to predict BMI from 2D shape and color PCs both separately and combined.

The naive elastic net performed best for the 2D shape-only and the color-only PC models, but for the combined 2D shape and color PC model the non-linear support vector machine performed significantly better than the elastic net (t(29) = 12.66, p < .001). The distribution of r^2^-values for the 30 repeats of each model are shown in Fig 3.

The mean amount of variance in BMI explained by 2D shape PCs alone was 23.4% and the mean amount of variance in BMI explained by color PCs alone was 27.2%. Combined, the 2D shape and color PCs explained 30.1% of the mean amount of variance in BMI.

The models for color-only PCs and 2D shape and color PCs combined were both significantly better than the model for 2D shape-only PCs (both t(58) > 12.86, both p < .001). The model for 2D shape and color PCs combined was additionally significantly better than the model for color-only PCs (t(58) = 8.66, p < .001).

### Comparing Principal Component and Facial Metric Models

Finally, we compared the three PC models (2D shape-only PCs, color-only PCs, and 2D shape and color PCs combined) with the four facial metric models (perimeter-to-area ratio only, width-to-height ratio only, cheek-to-jaw width only, and all 3 facial metrics combined). All PC models performed significantly better than all facial metric models (all t(58) > 37.2, all p < .001). For both facial metric and PC models we had complete separation between model training and model error assessment to avoid overfitting and thereby ensuring generalizable performance metrics.

## Discussion

All three of our PC models significantly outperformed each of the facial metric models, including a model that combined all three individual facial metrics. Among the three PC models, the non-linear 2D shape and color PC combined model significantly outperformed the linear 2D shape-only and color-only PC models. These results highlight the utility of a bottom up, data-driven approach to assessing BMI from face images.

While our PC models significantly outperformed existing facial metric models, comparison with results for correlations between perceptual ratings of women's facial adiposity and BMI suggest that they approach, but do not equal, the effectiveness of pooled adiposity ratings from human observers. For example, Tinlin *et al*. [3] report r^2^ values of .40, .44 and .46 for correlations between women's BMI and observers’ ratings of facial adiposity in three independent samples of faces.

There are several possible reasons human observers may outperform PC models. First and foremost, humans have encountered a much larger number of human body sizes and corresponding faces during their lifespan, which naturally makes them better trained at performing more complex cue integration than our models. Humans may also be capable of more fine-grained 2D shape discrimination than was captured by our 154-point 2D shape templates. It may be necessary to analyze 3D photographs to reach the accuracy of human raters. Lastly, the higher success of human raters could be caused by the possible presence of non-linear features in either shape or color face space, which would not be captured by our linear PCs and which humans could be trained to process and implement for assessing facial cues of adiposity. At this time, the contribution of nonlinear cues of adiposity to adiposity assessment from faces remains unknown, but analyses in the context of face recognition tasks suggest that the use of techniques to reduce nonlinear dimensionality can improve performance [32,33]. Thus, incorporating these techniques into analyses such as those performed in the current study may improve model performance. Importantly, each of our three PC models was closer to the level of performance of human observers than any of the facial metric models. This suggests that a PCA approach is a possible approximation to how humans assess faces.

Our results show that models using color information predicted BMI better than a model based on shape information alone. However, our face images were taken under carefully controlled photographic conditions in which color information was very carefully calibrated. Because shading holds information on 3D face shape and consequently also important cues to adiposity, differences in lightning will obstruct this shape information in uncontrolled photographic conditions. Therefore, models based on color PCs from less well-standardized face images may not outperform those based on 2D shape PCs. It is an open question whether the benefits of analyzing color information in our face images are entirely due to this information about 3D face shape or are also partly due to correlations between BMI and skin pigments. Indeed, recent research demonstrates that facial skin pigments do contain health information. For example, increased consumption of fruit and vegetables increases carotenoids in facial skin [34], suggesting facial skin pigments contain information about lifestyle health that may covary with BMI. Research using 3D face images may shed light on this issue by clarifying the extent to which color cues in 2D face images convey information about BMI through information about 3D facial morphology versus information about skin pigments.

In conclusion, we have here presented a data-driven approach for the prediction of BMI from 2D facial images and shown significant improvement on a previously reported top-down approach [8]. A support vector machine with a non-linear kernel and 2D shape and color principal components as predictors had the best performance for assessing BMI.

## Supporting Information

S1 Dataset Dataset for all participants. Files hold anonymized information on all participants for BMI, age, height, weight, facial metrics and shape and color principal components.
(ZIP)
