# Supplementary material for: Testing the Utility of a Data-Driven Approach for Assessing BMI from Face Images
Source: PLoS One. 2015 Oct 13;10(10):e0140347. doi: 10.1371/journal.pone.0140347 (PMC4603950; doi:10.1371/journal.pone.0140347)
Supplement: S1 Dataset — Files hold anonymized information on all participants for BMI, age, height, weight, facial metrics and shape and color principal components. (ZIP) [file pone.0140347.s001.zip › data/README.txt]

BMI_FM_PCs.txt holds information on all participants for BMI, age, height, weight, facial metrics and shape and color principal components. The principal components have here been generated from the entire data set and not inside a cross-validation, which can give some smaller fluctuations in results (most probably adding an upwards bias to performance) from this data set. 
